# Supplementary material for: Plant recruitment six years after the Samarco’s tailings-dam disaster: Impacts on species richness and plant growth
Source: PLoS One. 2025 Aug 25;20(8):e0315990. doi: 10.1371/journal.pone.0315990 (PMC12377569; doi:10.1371/journal.pone.0315990)
Supplement: Attachment — Submitted filename: Response to Reviewers.docx [file pone.0315990.s002.docx]

**June 05, 2025**

Dear Dr Marcela Pagano,
Academic Editor
PLOS ONE,

**RE: Manuscript ID PONE-D-24-55739**

Thank you for the opportunity to submit a revised version of our manuscript entitled “Plant recruitment six years after the Samarco’s tailings-dam disaster: Impacts on species richness and plant growth”.

We have carefully gone through the comments, so the new version of the manuscript can meet the journal’s requirements, and we have incorporated all the editorial corrections and the reviewer suggestions.

Thank you for your time and we look forward to hear from you in due course.

Florian Wittmann (corresponding author)

**Reviewers' comments:**

**Reviewer #1**: *The work is relevant and important, I suggest minor revisions, just the inclusion of photos of the collection sites, showing the conditions of the affected and unaffected environments, such as the state of the soil and the local scenery. As well as images of the plants found and the establishment of the plants.*

**RESPONSE:** We added three more images, as suggested by Reviewer #1. All figures were made according to PLoSOne specifications. They were uploaded and properly converted in the Preflight Analysis and Conversion Engine (PACE) digital diagnostic tool.

The figures and captions added are:

**Fig 2. Riverscapes of areas in the upper Doce River basin during the dry season.** Areas (A) affected by the Fundão dam disaster and (B) unaffected tributaries;

**Fig 3. Substrate samples from riverine banks spread in plastic trays for seedling emergence counting.** (A) Substrate from affected areas and (B) soil from unaffected tributaries.

**Fig 6. Growth habit and flowers from individuals of the growth experiment**. (A) Ludwigia octovalvis and (B) Marsypianthes chamaedrys.

We also added the respective citations of these figures in the preceding paragraph:

“Using similar geomorphology and vegetation types, we compared the floodplains of affected (A) rivers and unaffected (N) nearby tributaries (Fig 2).” (See page 6, line 75).

“In this way, we counted all plant individuals out of each plastic tray for six months (Fig 3).” (See page 7, line 111).

“Plants from both species flowered during the 75-day experiment in affected and unaffected substrates (Fig 6). All plants of *M. chamaedris* and *L. octovalvis* produced flowers.” (See page 17, line 294).

**Journal Requirements:**

1. *Please ensure that your manuscript meets PLOS ONE's style requirements, including those for file naming. The PLOS ONE style templates can be found at*

[*https://journals.plos.org/plosone/s/file?id=wjVg/PLOSOne_formatting_sample_main_body.pdf*](https://journals.plos.org/plosone/s/file?id=wjVg/PLOSOne_formatting_sample_main_body.pdf) *and*

[*https://journals.plos.org/plosone/s/file?id=ba62/PLOSOne_formatting_sample_title_authors_affiliations.pdf*](https://journals.plos.org/plosone/s/file?id=ba62/PLOSOne_formatting_sample_title_authors_affiliations.pdf)*.*

**RESPONSE:** We made the changes according to the templates and other information’s on the journal website.

a. On the first page, where each corresponding author was described, we changed the full name to the capitalized first letters of the name, after the respective email (according to the PDF, Title, Author, Affiliations, formatting guidelines):

* Corresponding Author:

Email: [**florian.wittmann@kit.edu**](mailto:florian.wittmann@kit.edu) (FW)

Email: [andrearaujodapaz@gmail.com](mailto:andrearaujodapaz@gmail.com) (AP)

b. We have changed the styles of the Headings of the three levels according to the rules.

c. Captions from Figures and Tables were updated to keep with the maximum word limit, bold type in the tilte, separated legends and specific location of captions in the text. The changed items and their new titles and legends are:

**Table 1: Chemical and nutritional parameters of soils used in the plant establishment and growth experiment.**

*S*oils from areas affected (A) and unaffected (N) by the tailings spill from the Fundão dam. SB is the sum of bases, t is the effective Cation Exchange Capacity, and T is the potential Cation Exchange Capacity at pH 7.0. Mean values and sample standard deviation (SD), from 9 samples of each treatment are presented. * Significant difference*.*

**Table 2: Species found in the soil seed bank of areas affected (A) or unaffected (N) by the passage of tailings from the *Fundão dam.***

^a^On the origin column, plants are classified as native or naturalized (Natur.).

^b^Seedling density is shown in the total number of emerging plants per m².

**Fig 4. Species richness and abundance of emerging plants in the seed bank experiment**. Sample-sized rarefaction curves (solid lines) and extrapolation (dashed lines) of the entire study. Curves from areas affected and unaffected by the passage of the tailings from the Fundão Dam, collected six years after the disaster. Species diversity based on the Hill numbers (q=0) and shaded areas represent 95% confidence intervals, which do not overlap (p<0.05).

**Fig 5. Variation in height and diameter of individuals from the study’s two most abundant species**. Vertical bars indicate the standard deviation of the mean. Different lower-case letters indicate significant differences in the parameters of the same species in time. *Significant overall difference for individuals of L. octovalvis plants.

**Fig 6. Growth habit and flowers from individuals of the growth experiment**. (A) Ludwigia octovalvis and (B) Marsypianthes chamaedrys.

2. *Please provide an amended statement that declares *all* the funding or sources of support (whether external or internal to your organization) received during this study, as detailed online in our guide for authors at* [*http://journals.plos.org/plosone/s/submit-now*](http://journals.plos.org/plosone/s/submit-now)*.  Please also include the statement “There was no additional external funding received for this study.” in your updated Funding Statement.*

*Please include your amended Funding Statement within your cover letter. We will change the online submission form on your behalf.*

**RESPONSE:** The text was updated as required and attached to the Cover Letter as follows:

“This work was supported by:

AP - CAPES, Coordenação de Aperfeiçoamento de Pessoal de Nível Superior (Processess 88882.437418/2019-01 and 88887.977524/2024-00) ([http://www.capes.gov.br](http://www.capes.gov.br/)) and DAAD, Deutscher Akademischer Austauschdienst (process 57507869) (<https://www.daad.de/de/>); CR and RC - CAPES, Coordenação de Aperfeiçoamento de Pessoal de Nível Superior ([http://www.capes.gov.br](http://www.capes.gov.br/)); FAPEMIG, Fundação de Amparo à Pesquisa do Estado de Minas Gerais (<http://www.fapemig.br/pt-br/>) and CNPq, Conselho Nacional de Desenvolvimento Científico e Tecnológico (<https://www.gov.br/cnpq/pt-br>); AW and FW – KIT, Karlsruhe Institute of Technology (<https://www.kit.edu/index.php>); AP, DA, AW, FW, CR and RC – FAPEMIG, Fundação de Amparo à Pesquisa do Estado de Minas Gerais and RENOVA* (number APQ-05461-18) (<http://www.fapemig.br/pt-br/> and <https://www.fundacaorenova.org/>); * This project/product was funded by the Renova Foundation, by imposition of the Transaction and Conduct Adjustment Term – TTAC, signed for the recovery, mitigation and compensation of the socioeconomic and socio-environmental impacts of the Fundão dam collapse, in Mariana, Minas Gerais.

We acknowledge support by the KIT-Publication Fund of the Karlsruhe Institute of Technology (https://www.bibliothek.kit.edu/english/kit-publication-fund-services.php).

The funders had no role in study design, data collection and analysis, decision to publish, or preparation of the manuscript. The authors declare no financial competing interests related to this study.

There was no additional external funding received for this study.”

3. *We note that your* ***Data Availability Statement*** *is currently as follows: [All relevant data are within the manuscript and its Supporting Information files]*

*Please confirm at this time whether or not your submission contains all raw data required to replicate the results of your study. Authors must share the “minimal data set” for their submission. PLOS defines the minimal data set to consist of the data required to replicate all study findings reported in the article, as well as related metadata and methods (*[*https://journals.plos.org/plosone/s/data-availability#loc-minimal-data-set-definition*](https://journals.plos.org/plosone/s/data-availability#loc-minimal-data-set-definition)*).*

*For example, authors should submit the following data:*

*- The values behind the means, standard deviations and other measures reported;*

*- The values used to build graphs;*

*- The points extracted from images for analysis.*

*Authors do not need to submit their entire data set if only a portion of the data was used in the reported study.*

*If your submission does not contain these data, please either upload them as Supporting Information files or deposit them to a stable, public repository and provide us with the relevant URLs, DOIs, or accession numbers. For a list of recommended repositories, please see* [*https://journals.plos.org/plosone/s/recommended-repositories*](https://journals.plos.org/plosone/s/recommended-repositories)*.*

*If there are ethical or legal restrictions on sharing a de-identified data set, please explain them in detail (e.g., data contain potentially sensitive information, data are owned by a third-party organization, etc.) and who has imposed them (e.g., an ethics committee). Please also provide contact information for a data access committee, ethics committee, or other institutional body to which data requests may be sent. If data are owned by a third party, please indicate how others may request data access.*

**RESPONSE:** All the data was made available at the Zenodo repository, by the following identifiers:

DOI: 10.5281/zenodo.15579256

<https://zenodo.org/records/15579256?token=eyJhbGciOiJIUzUxMiJ9.eyJpZCI6ImRlMWFlNTc0LTlmYmItNDIyNS1hNjRmLWI3YzBhZTRkMjE0MSIsImRhdGEiOnt9LCJyYW5kb20iOiIzNzdhMzJhYzQ5MDcxOWMxZGEzZmM4ZDAyYjAzM2VmZiJ9.9F5QYt5M1hkutgkan_nYHJKWiUhF2QYESHPLSiI89mD0EY1GdsX1iq0rfLu9_qyvSg2aSwj42drpdvoaVB1HvQ>

4. *We note that Figure 1 in your submission contain [map/satellite] images which may be copyrighted. All PLOS content is published under the Creative Commons Attribution License (CC BY 4.0), which means that the manuscript, images, and Supporting Information files will be freely available online, and any third party is permitted to access, download, copy, distribute, and use these materials in any way, even commercially, with proper attribution. For these reasons, we cannot publish previously copyrighted maps or satellite images created using proprietary data, such as Google software (Google Maps, Street View, and Earth). For more information, see our copyright guidelines:* [*http://journals.plos.org/plosone/s/licenses-and-copyright*](http://journals.plos.org/plosone/s/licenses-and-copyright)*.*

*We require you to either (1) present written permission from the copyright holder to publish these figures specifically under the CC BY 4.0 license, or (2) remove the figures from your submission:*

*a. You may seek permission from the original copyright holder of Figure 1 to publish the content specifically under the CC BY 4.0 license.*

*We recommend that you contact the original copyright holder with the Content Permission Form (*[*http://journals.plos.org/plosone/s/file?id=7c09/content-permission-form.pdf*](http://journals.plos.org/plosone/s/file?id=7c09/content-permission-form.pdf)*) and the following text:*

*“I request permission for the open-access journal PLOS ONE to publish XXX under the Creative Commons Attribution License (CCAL) CC BY 4.0 (*[*http://creativecommons.org/licenses/by/4.0/*](http://creativecommons.org/licenses/by/4.0/)*). Please be aware that this license allows unrestricted use and distribution, even commercially, by third parties. Please reply and provide explicit written permission to publish XXX under a CC BY license and complete the attached form.”*

*Please upload the completed Content Permission Form or other proof of granted permissions as an "Other" file with your submission.*

*In the figure caption of the copyrighted figure, please include the following text: “Reprinted from [ref] under a CC BY license, with permission from [name of publisher], original copyright [original copyright year].”*

*b. If you are unable to obtain permission from the original copyright holder to publish these figures under the CC BY 4.0 license or if the copyright holder’s requirements are incompatible with the CC BY 4.0 license, please either i) remove the figure or ii) supply a replacement figure that complies with the CC BY 4.0 license. Please check copyright information on all replacement figures and update the figure caption with source information. If applicable, please specify in the figure caption text when a figure is similar but not identical to the original image and is therefore for illustrative purposes only.*

*The following resources for replacing copyrighted map figures may be helpful:*

*USGS National Map Viewer (public domain):* [*http://viewer.nationalmap.gov/viewer/*](http://viewer.nationalmap.gov/viewer/)

*The Gateway to Astronaut Photography of Earth (public domain):* [*http://eol.jsc.nasa.gov/sseop/clickmap/*](http://eol.jsc.nasa.gov/sseop/clickmap/)

*Maps at the CIA (public domain):* [*https://www.cia.gov/library/publications/the-world-factbook/index.html*](https://www.cia.gov/library/publications/the-world-factbook/index.html) *and* [*https://www.cia.gov/library/publications/cia-maps-publications/index.html*](https://www.cia.gov/library/publications/cia-maps-publications/index.html)

*NASA Earth Observatory (public domain):* [*http://earthobservatory.nasa.gov/*](http://earthobservatory.nasa.gov/)

*Landsat:* [*http://landsat.visibleearth.nasa.gov/*](http://landsat.visibleearth.nasa.gov/)

*USGS EROS (Earth Resources Observatory and Science (EROS) Center) (public domain):* [*http://eros.usgs.gov/#*](http://eros.usgs.gov/)

*Natural Earth (public domain):* [*http://www.naturalearthdata.com/*](http://www.naturalearthdata.com/)

**RESPONSE:** Figure 1 was changed to meet the specifications. The satellite image was replaced, using the suggested OpenStreetMap web site, which is open source.

The final figure was uploaded to Preflight Analysis and Conversion Engine (PACE) digital diagnostic tool to assure that the journal requirements are met.

This is the caption for Fig1:

**”Fig 1. Location of the areas sampled in the three regions of the study.** Above, from left to right: South America, Brazil, and the state of Minas Gerais. Bellow: Sampling carried out in the regions: 1) downstream of the Fundão dam, 2) downstream of Paracatu de Baixo and 3) upstream of the Risoleta Neves Hydroelectric Power Plant. The tailings deposition zone is highlighted along the sampled region (shape file “PG23_Area_Afetada_Lama” provided by Fundação RENOVA). Map created using QGIS 3.34.14. Map data from openstreetmap.org/copyright.” (See page 5, lines 63-64).

The following text also related to the map was added to the Materials and methods section:

“Map was created using software QGIS 3.34.14 [25].”

5. *We are unable to open your Supporting Information file. Please kindly revise as necessary and re-upload.*

**RESPONSE:** All the data is now available in the Zenodo repository, so there will be no Supporting Information file. The information is available using the DOI: 10.5281/zenodo.15579256

6. *Please review your reference list to ensure that it is complete and correct. If you have cited papers that have been retracted, please include the rationale for doing so in the manuscript text or remove these references and replace them with relevant current references. Any changes to the reference list should be mentioned in the rebuttal letter that accompanies your revised manuscript. If you need to cite a retracted article, indicate the article’s retracted status in the References list and also include a citation and full reference for the retraction notice.*

**RESPONSE:** In the submitted manuscript some references were cited as texts and not as numbers, due to problems in the reference management program used. The following references were updated in each section:

**Introduction** – (Samarco Mineração S.A. 2016; IBAMA 2020) became [10 and 11]; (Schupp 1995) became [12] since was previously cited as text and wasn’t present in the Reference List; the number of the following references citations were changed because of this; (Silva et al. 2021) was cited as text in the first time and was kept as [16].

**Materials and methods** - (Morellato, 2016) became [26]; Mesgaran et al. (2007), already present in the list, became [27]; The website (http://floradobrasil.jbrj.gov.br/) became [33]; (Souza 2019) became [36]; (Hashimoto 2020) became [37]. (EMBRAPA 2017) became [35]

**Discussion** - (see Silva et al. 2021) was updated to [16] and one more citation was added, already referenced as [21]; Another article with the same author’s surname, Silva et al. 2021, was corrected to [43]; (Barret, 1992) wasn’t in the reference list and became [44]. (Silva et al 2021) was updated to [16]; (Souza 2019) became [36]. Amorim 2021 became [48].

The cited references that were previously only present in the main text, were updated to the reference list as follows:

[12] Schupp EW. Seed-Seedling Conflicts, Habitat Choice, and Patterns of Plant Recruitment. Am J Bot 1995;82:399–409.

[26] Morellato LPC, Alberton B, Alvarado ST, Borges B, Buisson E, Camargo MGG, et al. Linking plant phenology to conservation biology. Biol Conserv 2016;195:60–72. https://doi.org/10.1016/j.biocon.2015.12.033.

[33] Flora e Funga do Brasil. Jardim Botânico do Rio de Janeiro 2023. Available from: http://floradobrasil.jbrj.gov.br/ (accessed June 7, 2023).

[36] Souza NXM, Vieira AOS, Costa GM, Aona LYS. Diagnostic characters important for the identification of species of Ludwigia (Onagraceae) from the Recôncavo basin of Bahia, Brazil. Rodriguesia 2019;70. https://doi.org/10.1590/2175-7860201970085.

[37] Hashimoto MY, Ferreira HD. Taxonomic study of marsypianthes mart. Ex benth. (hyptidinae, lamiaceae) in brazil. Acta Bot Brasilica 2020;34:277–89. https://doi.org/10.1590/0102-33062019abb0339.

[35] EMBRAPA (Empresa Brasileira de Pesquisa Agropecuária). Manual de Métodos de Análise de Solo. 2^a^ Ed. Rio de Janeiro: Ministério da Agricultura e do Abastecimento; 1997

[43] Silva CV, Martins SV, Villa PM, Correa Kruschewsky G, Dias AA, Nabeta FH. Seed bank of mining tailings as indicator of vegetation regeneration in mariana, brazil. Rev Biol Trop 2021;69:700–16. <https://doi.org/10.15517/rbt.v69i2.41800>.

[44] Barrett JP, Silander JA. Seedling Recruitment Limitation in White Clover (Trifolium repens; Leguminosae). vol. 79. 1992.

[48] Amorim T, Santos AMM, Castro CC, Leite AV. The explosive flowers of Marsypianthes chamaedrys (Lamiaceae) resort to self-pollination and improve reproduction. Acta Bot Brasilica 2022;35:707–13. https://doi.org/10.1590/0102-33062020abb0478.
