# Supplementary material for: Plant recruitment six years after the Samarco’s tailings-dam disaster: Impacts on species richness and plant growth
Source: PLoS One. 2025 Aug 25;20(8):e0315990. doi: 10.1371/journal.pone.0315990 (PMC12377569; doi:10.1371/journal.pone.0315990)
Supplement: Attachment — Submitted filename: Response_to_Reviewers_auresp_2.docx [file pone.0315990.s003.docx]

**August 03, 2025**

Dear Dr Marcela Pagano,
Academic Editor
PLOS ONE,

**RE: Manuscript ID PONE-D-24-55739**

Thank you for the opportunity to submit a revised version of our manuscript entitled “Plant recruitment six years after the Samarco’s tailings-dam disaster: Impacts on species richness and plant growth”.

We have carefully gone through the manuscript, checking for any SI units-related corrections needed. We also looked for other minor alterations, which are marked in the following response description. All the references were rechecked, and we made the respective alterations following the journal's criteria.

Thank you again for your time, and we look forward to hearing from you in due course.

Florian Wittmann (corresponding author)

**Reviewer comment:**

*“Dear author, please check for using SI units, such as h, d, etc.”*

**RESPONSE:**

We have revised the manuscript and made the changes according to the International Systems of Units (SI) as defined in the 9^th^ edition (2019, updated 2024; <https://www.bipm.org/en/publications/si-brochure/>). We also made additional adjustments to align with the journal’s requirements, related to formatting in the text and the reference list.

1. The word “day” was replaced by “d” whenever needed, or removed when not strictly necessary:

Line 168: “These plants were kept in a greenhouse for 75 d~~ays~~, …”

Line 271: The growth experiment ~~over 75 days~~ revealed…”

Line 273: “…specially after 60 d~~ays~~ ...”

Line 277: “…in the increment in height and diameter over 75 d~~ays~~ comparing …“

Line 280: “…plants continued to grow with similar rate after 75 d~~ays~~…”

Lines 294: “Plants from both species flowered during the ~~75-day~~ experiment…”

2. The word “hour” was replaced by “h”:

Line 108: “… with a photoperiod of 12 h”

3. The word “meters” was replaced by “m”:

Line 97: “…we selected 5 points at a minimum distance of 300 m from each other,…”

4. To maintain consistency, the references to concentration units were standardized, using the '/' symbol instead of exponents (negative powers):

Lines 28 and 29: “For instance, the total contents of Cr (235.0 ± 69.5 mg/kg) and Pb (52.7 ± 15.9 mg/kg) were higher than the soil quality guideline values, which are 75 and 19.5 mg/kg, respectively.“

5. The litre symbol was changed to capital letter “L”, as suggested in the Si document: “to avoid the risk of confusion between the letter l (el) and the numeral 1 (one)”:

Line 167: “we homogenized the soil and divided it into 500 mL pots”

6. The correct lowercase form of “kg” replaced the capital letter “Kg” in Table 1:

Lines 224 and 225: Org. Mat. (dag/kg)

7. The symbol “×” replaced the letter “x” for multiplicative purposes:

Line 106: “We transferred the sampled soils to plastic trays (0.25 m × 0.5 m × 0.1 m high)“

Line 99: “At each point, we collected the topsoil and the litterfall using a metal jig (25 × 25 cm) at 5 cm soil depth.”

8. The space that preceded the temperature symbol “°C” was removed:

Line 107: “…with controlled conditions at 25 (± 2)°C, 70% “

Line 140: “We kept the soil samples in plastic bags at 15°C and transported…”

Line 173: “…weighed on an analytical balance and dried in an oven at 60°C to a constant weight.”

9. One of the “°C” had the wrong symbol, and the masculine ordinal indicator “º” was replaced by the correct degree symbol “°”:

Line 173: “…weighed on an analytical balance and dried in an oven at 60°C ...”

10. Commas that were previously used for decimals were replaced by a period

Line 157: “being an amphibious plant, between 0.3 to 2.5 m tall”

Line 323: “Furthermore, in our experiment, 693.3 individuals/m² emerged in the affected areas.“

11. In order to keep abbreviations to a minimum, two acronyms were removed due to little usage in the manuscript. “PTE”, for potentially toxic elements, is mentioned in full and “DGL”, for diameter at ground level, is cited as just “diameter” after first appearance:

Line 39: “…and accumulation of potentially toxic elements.”

Line 169:” … and diameter at ground level every fortnight.”

12. The font size in Table 1 was standardized, since some symbols were one unit smaller:

Lines 224 and 225: See parameters P, K, Na, Org. mat, P-Rem, Cu, Mn, Fe, Zn, Cr, Ni, Cd, and Pb.

13. For clarification, the word “mean” was added in the parentheses:

Line 287:” the root:shoot ratio (mean ± SD) in plants growing“

14. Typographical errors, such as the lack of a letter or not translated word, were corrected:

Line 302: “We also showed that soil affected by Samarco slurry reduced plant growth “

Line 307: “which shows that Samarco-slurry does not prevent plant regeneration”

Line 314 and 315: “On the other hand, the unaffected areas had higher levels of: Cu, Fe, Zn, Cr and Ni, possibly due to previous long-term anthropogenic activities…”

Line 377: “but still shows that Samarco-slurry does not prevent plant regeneration.”

15. Names in Portuguese that were in italics were changed to non-italicized format for standardization, following other papers published in this journal:

Title: “Plant recruitment six years after the Samarco’s tailings-dam disaster: Impacts on species richness and plant growth”

Abstract: “… when a dam from the mining company Samarco was breached. Millions of mine tailings from this upstream embankment were dumped over the Doce River basin …”,

Keywords: “Rio Doce”

Line 6: “…when the Fundão dam (Samarco’s Company),…”

Line 7: “…slurry were released into the Doce River basin…”

Lines 23 and 24: “Furthermore, in the affected banks of the Doce river tributary, Gualaxo do Norte, …”

Line 43: “…six years after the Samarco dam breach…”

Line 51: “We sampled the Upper Doce River basin in Minas Gerais in southeast Brazil “

Line 60 and 61: “1) downstream of the Fundão dam, 2) downstream of Paracatu de Baixo and 3) upstream of the Risoleta Neves Hydroelectric Power Plant”

Line 63: “…provided by Fundação RENOVA”

Lines 68 and 69: “…composing the slurry pathway, from the Fundão dam to the Risoleta Neves HPP”

Line 72: “The areas near Fundão dam have a history of mining activity…”

Line 78: “Riverscapes of areas in the upper Doce River basin during the dry season”

Line 83: “We sampled soils for the seed bank experiment in three regions between the *Fundão* dam and the Risoleta Neves HPP”

Lines 84 to 88: “. A1) Gualaxo do Norte River, close to Fundão Dam, with mostly forested floodplains (20º 15’ 13’’S, 43 º 25’ 16’’W); A2) Gualaxo do Norte River, near Paracatu de Baixo, with fragments of Atlantic forest mixed with pasture (20º 17’ 43’’S, 43º 11’ 51’’W); and A3) Doce River, upstream of the HPP, in a mainly farming and livestock landscape with less forest fragments (20º 14’ 10’’S, 42º 53’ 05’’W).”

Lines 89 to 92: “Additionally, in the unaffected areas, we sampled the riparian zone of non-affected tributaries, with characteristics similar to those of the affected areas. N1) Gualaxo do Norte River (20º 16’ 32’’S, 43 º 26’ 00’’W); N2) Bucão stream (20º 16’ 06’’S, 43º 12’ 36’’W); and N3: Piranga River (20º 19’ 28’’S, 42º 53’ 49’’W) (Fig 1).”

Line 126: “To verify whether the species are native or not, thinking about their usage in future restoration projects, we used the Flora e Funga do Brasil website [33] to classify them as native or naturalized [34]. “

Line 133: “Those soil samples were taken from one region around the affected HPP’s lake (20º 14’ 10’’S, 42º 53’ 05’’W), and in the banks of the Piranga River, the closest unaffected tributary near the reservoir “

Line 143: “The soil was divided to obtain subsamples for chemical analysis at the Soil Physics Laboratory of the Federal University of Viçosa - UFV [35]. “

Line 236: “Table 2: Species found in the soil seed bank of areas affected (A) or unaffected (N) by the passage of tailings from the Fundão dam”

Line 255: “Curves from areas affected and unaffected by the passage of the tailings from the *Fundão* Dam, collected six years after the disaster.”

Line 392: “…leadership and support at Terra Água and Macroflora research groups”

16. In the reference list, the citations were updated to meet journal requirements: we corrected journal abbreviations, changed lowercase and uppercase letters when needed, standardized font formatting, wrote species names in italics, replaced “https://doi.org/” with “doi:”, and replaced “accessed” with “cited” on internet references. The font type Arial, used throughout the manuscript, was also used to standardize all the references in the list.

Reference 1:

Tarolli P, Sofia G. Human topographic signatures and derived geomorphic processes across landscapes. Geomorphology (Amst) 2016;255:140–61. doi:10.1016/j.geomorph.2015.12.007.

Reference 2:

Dong L, Deng S, Wang F. Some developments and new insights for environmental sustainability and disaster control of tailings dam. J Clean Prod 2020;269:122270. doi:10.1016/j.jclepro.2020.122270.

Reference 3:

Meira-Neto JAA, Neri AV. Appealing the death sentences of the Doce, São Francisco and Amazonas rivers: stopping the Mining Lobby and creating ecosystem services reserves. Perspect Ecol Conserv 2017;15:199–201. doi:10.1016/j.pecon.2017.06.008.

Reference 4:

Sánchez LE, Alger K, Alonso L, Francisco B, Brito MC, Laureano F, et al. Impacts of the Fundão Dam failure: a pathway to sustainable and resilient mitigation. Rio Doce Panel Thematic Report 2018;1:1–52. doi:10.2305/iucn.ch.2018.18.en.

Reference 5:

Fernandes GW, Goulart FF, Ranieri BD, Coelho MS, Dales K, Boesche N, et al. Deep into the mud: ecological and socio-economic impacts of the dam breach in Mariana, Brazil. Perspect Ecol Conserv 2016;14:35–45. doi:10.1016/j.ncon.2016.10.003.

Reference 6:

Walck JL, Baskin JM, Baskin CC, Hidayati SN. Defining transient and persistent seed banks in species with pronounced seasonal dormancy and germination patterns. Seed Sci Res 2005;15:189–96. doi:10.1079/ssr2005209.

Reference 7:

Nazareno AG, Knowles LL, Dick CW, Lohmann LG. By animal, water, or wind: can dispersal mode predict genetic connectivity in riverine plant species? Front Plant Sci 2021;12:1–18. doi:10.3389/fpls.2021.626405.

Reference 8:

Campanharo ÍF, Martins S V., Villa PM, Kruschewsky GC, Dias AA, Nabeta FH. Forest restoration methods, seasonality, and penetration resistance does not influence aboveground biomass stock on mining tailings in Mariana, Brazil. An Acad Bras Cienc 2021;93. doi:10.1590/0001-3765202120201209.

Reference 9:

Doelinger TM. Atualização do Plano de Recuperação Ambiental Integrado - PRAI [Internet] 2017:1–139. Available from: https://www.reparacaobaciariodoce.com/wp-content/uploads/2025/01/PRAI_renova_jan17_rev03.pdf (cited August 24, 2023).

Reference 10:

Orlandini D. Contenção de carreamento de sedimentos por meio de revegetação [Internet]. Samarco Mineração SA 2016:1–61. Available from: https://www.ibama.gov.br/phocadownload/emergenciasambientais/anexo_v_relatorio_revegetao_agroflor.pdf (cited August 27, 2023).

Reference 11:

Fonseca Ê. Nota Técnica N^o^ 4/2020/CT-FLOR/GABIN [Internet] 2020:1–7. Available from: https://www.gov.br/ibama/pt-br/acesso-a-informacao/institucional/cif/arquivos/notas-tecnicas/CT-FLOR/2020/cif-nt-ct-flor-04-2020.pdf (cited August 24, 2023).

Reference 13:

Larson JE, Ebinger KR, Suding KN. Water the odds? Spring rainfall and emergence-related seed traits drive plant recruitment. Oikos 2021;130:1665–78. https://doi.org/doi:10.1111/oik.08638.

Reference 14:

Queiroz HM, Ferreira AD, Ruiz F, Bovi RC, Deng Y, de Souza Júnior VS, et al. Early pedogenesis of anthropogenic soils produced by the world’s largest mining disaster, the “Fundão” dam collapse, in southeast Brazil. Catena (Amst) 2022;219. https://doi.org/doi:10.1016/j.catena.2022.106625.

Reference 15:

Andrade GF, Paniz FP, Martins AC, Rocha BA, da Silva Lobato AK, Rodrigues JL, et al. Agricultural use of Samarco’s spilled mud assessed by rice cultivation: A promising residue use? Chemosphere 2018;193:892–902. https://doi.org/doi:10.1016/j.chemosphere.2017.11.099.

Reference 16:

Silva AO, Guimarães AA, Lopez BDO, Zanchi CS, Vega CFP, Batista ÉR, et al. Chemical, physical, and biological attributes in soils affected by deposition of iron ore tailings from the Fundão Dam failure. Environ Monit Assess 2021;193:18. doi:10.1007/s10661-021-09234-4.

Reference 17:

Bruno D, Gutiérrez-Cánovas C, Sánchez-Fernández D, Velasco J, Nilsson C. Impacts of environmental filters on functional redundancy in riparian vegetation. J Appl Ecol 2016;53:846–55. doi:10.1111/1365-2664.12619.

Reference 19:

Cruz FV da S, Gomes MP, Bicalho EM, Della Torre F, Garcia QS. Does Samarco’s spilled mud impair the growth of native trees of the Atlantic Rainforest? Ecotoxicol Environ Saf 2020;189:110021. doi:10.1016/j.ecoenv.2019.110021.

Reference 20 :

Nascimento F dos S, Toledo AMO, Pimenta M de P, Resende CF de, Peixoto PHP, Zimerer A, et al. Does mining waste concentration in the soil interfere with leaf selection by Acromyrmex subterraneus (Formicidae)? Biotropica 2021;53:487–95. doi:10.1111/btp.12892.

Reference 21:

Coelho DG, Marinato CS, de Matos LP, de Andrade HM, da Silva VM, Neves PHS, et al. Evaluation of Metals in Soil and Tissues of Economic-Interest Plants Grown in Sites Affected by the Fundão Dam Failure in Mariana, Brazil. Integr Environ Assess Manag 2020. doi:10.1002/ieam.4253.

Reference 22:

Instituto Brasileiro de Geografia e Estatística. Manual Técnico da Vegetação Brasileira. vol. 1. 2nd ed. Rio de Janeiro: Instituto Brasileiro de Geografia e Estatística; 2012.

Reference 23:

Coelho ALN. Bacia hidrográfica do Rio Doce (MG/ES): uma análise socioambiental integrada. Geografares 2009;7:131–46.

Reference 25:

QGIS Development Team. QGIS Geographic Information System [Internet]. Available from: Http://QgisOsgeoOrg 2024.

Reference 26:

Morellato LPC, Alberton B, Alvarado ST, Borges B, Buisson E, Camargo MGG, et al. Linking plant phenology to conservation biology. Biol Conserv 2016;195:60–72. doi:10.1016/j.biocon.2015.12.033.

Reference 27:

Mesgaran MB, Mashhadi HR, Zand E, Alizadeh HM. Comparison of three methodologies for efficient seed extraction in studies of soil weed seedbanks. Weed Res 2007;47:472–8. doi:10.1111/j.1365-3180.2007.00592.x.

Reference 32:

Piaia BB, Rovedder APM, Piazza EM, Stefanello M de M, Felker RM, Costa EA. Floristic composition analysis of soil transposition in a seasonal forest in Rio Grande do Sul, Brazil. Floresta e Ambiente 2019;26. https://doi.org/doi:10.1590/2179-8087.016317.

Reference 33:

Flora e Funga do Brasil. Jardim Botânico do Rio de Janeiro [Internet] 2023. Available from: http://floradobrasil.jbrj.gov.br/ (cited June 7, 2023).

Reference 34:

Richardson DM, Pyšek P, Rejmánek M, Barbour MG, Dane Panetta F, West CJ. Naturalization and invasion of alien plants: Concepts and definitions. Divers Distrib 2000;6:93–107. doi:10.1046/j.1472-4642.2000.00083.x.

Reference 36:

Souza NXM, Vieira AOS, Costa GM, Aona LYS. Diagnostic characters important for the identification of species of Ludwigia (Onagraceae) from the Recôncavo basin of Bahia, Brazil. Rodriguesia 2019;70. doi:10.1590/2175-7860201970085.

Reference 37:

Hashimoto MY, Ferreira HD. Taxonomic study of *Marsypianthes* Mart. ex Benth. (Hyptidinae, Lamiaceae) in Brazil. Acta Bot Brasilica 2020;34:277–89. doi:10.1590/0102-33062019abb0339.

Reference 38:

Sainju UM, Allen BL, Lenssen AW, Ghimire RP. Root biomass, root/shoot ratio, and soil water content under perennial grasses with different nitrogen rates. Field Crops Res 2017;210:183–91. doi:10.1016/j.fcr.2017.05.029.

Reference 39:

Chao A, Gotelli NJ, Hsieh TC, Sander EL, Ma KH, Colwell RK, et al. Rarefaction and extrapolation with Hill numbers: a framework for sampling and estimation in species diversity studies. Ecol Monogr 2014;84:45–67. doi:10.1890/13-0133.1.

Reference 42:

Davila RB, Fontes MPF, Pacheco AA, Ferreira M da S. Heavy metals in iron ore tailings and floodplain soils affected by the Samarco dam collapse in Brazil. Sci Total Environ 2020;709. doi:10.1016/j.scitotenv.2019.136151.

Reference 43:

Silva CV, Martins SV, Villa PM, Correa Kruschewsky G, Dias AA, Nabeta FH. Seed bank of mining tailings as indicator of vegetation regeneration in Mariana, Brazil. Rev Biol Trop 2021;69:700–16. doi:10.15517/rbt.v69i2.41800.

Reference 44:

Barrett JP, Silander JA. Seedling Recruitment Limitation in White Clover (*Trifolium repens*; Leguminosae). Am J Bot 1992; 79:643-649.

Reference 45:

Marques AR, Atman APF, Silveira FAO, de Lemos-Filho JP. Are seed germination and ecological breadth associated? Testing the regeneration niche hypothesis with bromeliads in a heterogeneous neotropical montane vegetation. Plant Ecol 2014;215:517–29. doi:10.1007/s11258-014-0320-4.

Reference 47:

Soares MBB, Bianco S, Finoto EL, Bolonhezi D, Albuquerque JAA. Phytosociological study on the weed communities in green sugarcane field reform using conservation tillage and oilseed crops in succession. Appl Ecol Environ Res 2017;15:417–28. doi:10.15666/aeer/1503_417428.

Reference 48:

Amorim T, Santos AMM, Castro CC, Leite AV. The explosive flowers of Marsypianthes chamaedrys (Lamiaceae) resort to self-pollination and improve reproduction. Acta Bot Brasilica 2022;35:707–13. doi:10.1590/0102-33062020abb0478.

Reference 49:

Jordão TC, Prado IG de O, da Silva M de CS, Diogo NV, Prates Júnior P, Veloso TGR, et al. Shifts in Arbuscular Mycorrhizal fungal properties due to vegetative remediation of mine spoil contamination from a dam rupture in Mariana, Brazil. Applied Soil Ecology 2021;162. doi:10.1016/j.apsoil.2021.103885.

Reference 50:

Cui X, Geng Y, Li T, Zhao R, Li XX, Cui Z. Field application and effect evaluation of different iron tailings soil utilization technologies. Resour Conserv Recycl 2021;173. doi:10.1016/j.resconrec.2021.105746.
